# Dementia-specific risks of scabies: Retrospective epidemiologic analysis of an unveiled nosocomial outbreak in Japan from 1989–90

**DOI:** 10.1186/1471-2334-5-85

**Published:** 2005-10-14

**Authors:** Masae Tsutsumi, Hiroshi Nishiura, Toshio Kobayashi

**Affiliations:** 1 School of Nursing, Yamaguchi Prefectural University, Miyanoshimo, Yamaguchi, Japan; 2 Graduate School of Health Sciences, Hiroshima University, Kasumi 1-2-3, Minamiku, Hiroshima, Japan; 3 Department of Medical Biometry, University of Tübingen, Westbahnhofstr. 55-D, 72070, Tübingen, Germany

## Abstract

**Background:**

Although senile dementia patients in long-term care facilities are at leading risk of scabies, the epidemiologic characteristics of this disease have yet to be fully clarified. This study documents the findings of a ward-scale nosocomial outbreak in western Japan from 1989–90, for which permission to publish was only recently obtained.

**Methods:**

A retrospective epidemiologic study was performed to identify specific risk factors of scabies among patients with dementia. Analyses were based on a review of medical and nursing records. All inpatients in the affected ward at the time of the outbreak were included in the study. Observational and analytical approaches were employed to assess the findings.

**Results:**

Twenty of 65 inpatients in the ward met the case definition of scabies. The outbreak lasted for almost 10 months and as a result, the spatial distribution of infections showed no localized patterns in the latter phase of the outbreak. The duration of illness significantly decreased after initiation of control measures (P = 0.0067). Movement without assistance (Odds Ratio [OR] = 11.3; 95% Confidence Interval [CI]: 2.9, 44.8) and moving beyond the room (but within the ward) (OR = 4.1; 95% CI: 1.4, 12.5) were significantly associated with infection, while types of room (Western or Japanese) and sleeping arrangement (on beds or futons laid directly on the floor) appeared not to be risk factors.

**Conclusion:**

Univariate analysis demonstrated the importance of patients' behaviours during daily activities in controlling scabies among senile dementia patients. The findings also support previous evidence that catching scabies from fomites is far less common. Moreover, since cognitive disorders make it difficult for individuals to communicate and understand the implications of risky contacts as well as treatment method, and given the non-specific nature of individual contacts that are often unpredictable, real-time observations might help improve control practices.

## Background

Scabies is a contagious skin irritation caused by the small translucent mite *Sarcoptes scabiei*(itch mite). Allergic responses to these mites and the waste products they produce lead to development of extensive areas of inflamed, reddened itchy skin [1]. The disease is transmitted from person to person by direct skin contact [2] and continues to be a major problem in nursing homes in industrialized countries, particularly among debilitated patients who require extensive hands-on care [3]. The clinical features of scabies in the elderly differ from those in younger individuals and such episodes are often the cause of nosocomial outbreaks because of delayed diagnosis due to the inspecificity of the lesions [4]. This is especially true among elderly individuals diagnosed with senile, psychogenic or degenerative diseases and unable to directly complain of their symptoms. A lack of attention to individual protection measures by healthcare workers (HCWs) has also been described as a cause of delayed diagnosis [4].

Even though several reports have documented local outbreaks and dermatological case descriptions, these remain insufficient in helping identify the epidemiologic characteristics of nosocomial outbreaks. Particularly, hospital-based epidemiologic investigations focusing on patterns of transmission not only among caregivers but also among elderly inpatients are necessary in establishing and activating an appropriate surveillance system. The specific trends of a scabies outbreak were previously observed in a geriatrics hospital in Japan from 1989–90 (Tsutsumi M, unpublished data). Although these observations were neither announced nor reported officially because of factors related to the reputation of the hospital, we recently obtained permission to study and report the epidemiologic details. This paper describes a ward-scale outbreak of scabies among elderly inpatients with senile dementia in an attempt to characterize the risk factors and patterns of spread of infection through a retrospective epidemiologic study based on unveiled outbreak records.

## Methods

### The outbreak

On 6 May 1989, an 85-year-old female patient with senile dementia presented with tiny red dots and surrounding skin redness on her abdomen and both femoral regions, and was consequently diagnosed with scabies. She was housed in a dementia ward in a 435-bed geriatrics hospital in western Japan. The hospital was equipped with specially authorized geriatric wards according to Japanese law. Diagnoses of scabies in the dementia ward continued until 7 December 1989. Preventive measures were not instituted until 4 months after diagnosis of the index case and no prophylactic treatment of uninfected inpatients or HCWs was performed throughout; staff awareness of and adherence to infection control practice seem to have been insufficient at this time.

### Case definition and diagnosis

All inpatients in the dementia ward were diagnosed with senile dementia due to prior cerebrovascular or degenerative diseases. Suspected cases of scabies in this study were defined as persons 1) housed in the dementia ward and 2) who presented with clinical signs (generalized or localized pruritus of several days evolution or appearance of cutaneous lesions suggesting scabies regardless of their severity and extent) during the outbreak period (May 1989 to February 1990). Confirmed diagnoses were made by dermatologists through direct bedside microscopic examinations of *Sarcoptes scabiei*. Since there was no attending dermatologist in the hospital, dermatologists working part-time once a week under the support of an outpatient service conducted these consultations.

### Study background (observational study)

The aim of this study was to identify specific features of scabies outbreaks in dementia wards. Although the hospital authority in question previously prohibited documentation of the outbreak, detailed clinical and epidemiologic information was obtained by the first author (MT) for academic purpose while working as a nurse in this institute. In addition to the data obtained through personal observations, clinical information was retrospectively obtained by reviewing medical and nursing records of inpatients housed in the ward during the outbreak period.

### Exposure and statistical analysis

To assess the association between dementia ward-specific characteristics and scabies, we conducted a retrospective study of all inpatients in the ward during the outbreak period. Investigated dichotomous variables were as follows: 1) individual demographic characteristics (gender); 2) type of room (Western-type floor made of wood or traditional Japanese tatami mats made of straw); 3) sleeping arrangement (on a bed or futon laid directly on the floor); 4) ease of movement (need for assistance); and 5) range of movement (confined to the room or ward). Age was not included in the analyses since the ward in question only housed patients with senile dementia. Although multivariate analyses could not be conducted due to the lack of information on non-infected individuals, stored data based on hospital records enabled investigation of univariate associations.

Comparisons between groups were performed using Fisher's exact test to assess the univariate association between the investigated variables and infection. The level of statistical significance was set at P = 0.05. Comparisons of the duration of illness before and after the initiation of control measures were performed using the F-test followed by the Student t-test or Welch test. Duration was compared between those diagnosed before 30 Sep 1989 (n = 6) and those diagnosed thereafter (n = 15). All data entry was performed by two different persons using Microsoft Excel 2000 (Microsoft Corporation, Redmond, WA) and double-checked. The statistical data were analysed using the statistical software R (R Development Core Team, Vienna) [5] and JMP IN ver. 5.1 (SAS Institute Inc., Cary, NC).

## Results

### Personal characteristics

Twenty of the 65 dementia ward inpatients (30.8%; 18 females, 2 males) developed symptoms. The mean age at infection was 81.6 years (standard deviation (SD) = 7.9; median = 80.5). The chain of nosocomial transmission was observed only within the dementia ward investigated. Detailed descriptions of each case are provided in Additional file 1.

### Temporal and spatial distribution

**Figure 1:**
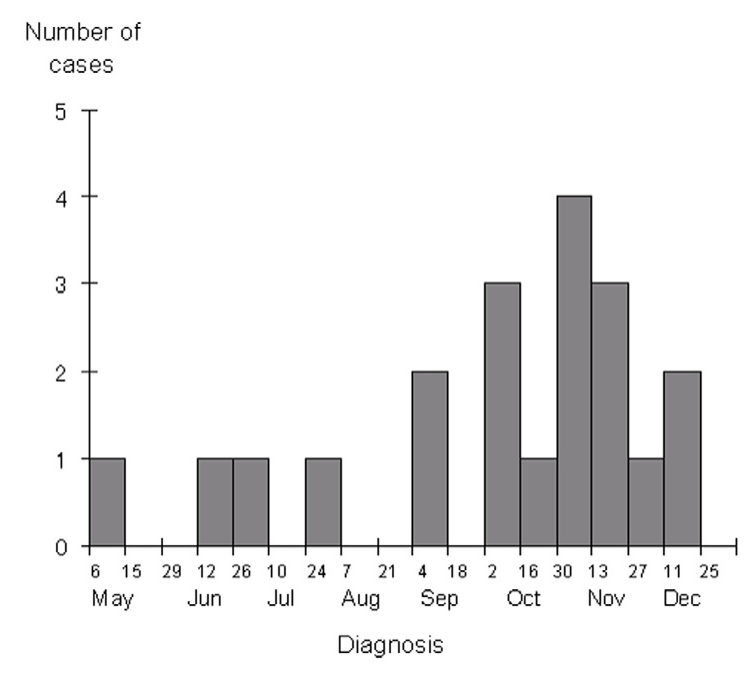
**Temporal distribution of the scabies cases (n = 20) according to the time of diagnosis**. Biweekly numbers of cases diagnosed on the dates given are shown.

**Figure 2:**
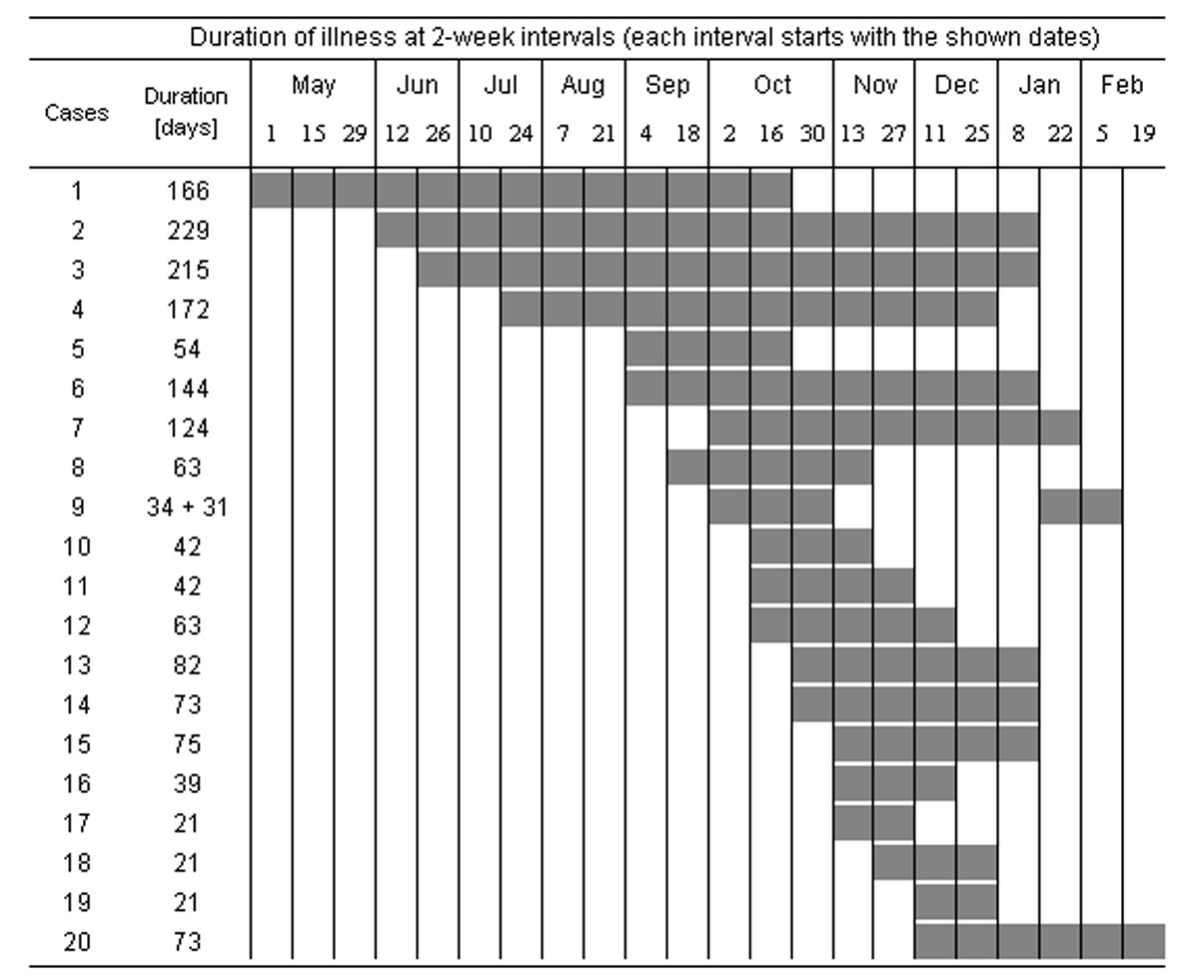
**Duration of illness of the scabies cases**. Duration is shown at two-week intervals. Note: illness onset was biased by the delay in diagnosis.

The outbreak continued for a total of 288 days. The epidemic curve peaked in early November (Figure 1) with 15 patients showing associated clinical signs in mid-November (Figure 2). None of the ward staff were reported as having scabies and no cases were reported among private individuals (i.e., relatives of patients).

**Figure 3:**
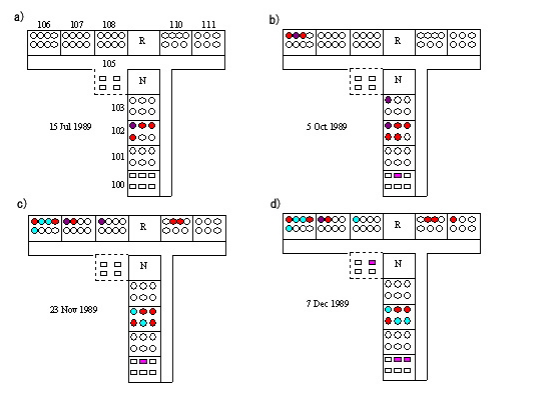
**Spatial distribution of the scabies cases (n = 20) with time**. Ward-scale spatial distribution of the scabies cases are shown as of a) 15 July, b) 5 October, c) 23 November and d) 7 December, 1989. Circles and squares denote inpatients sleeping on futons directly on tatami flooring and beds, respectively. Red denotes individuals able to move without assistance and beyond their room, purple denotes those able to move without assistance but only within their room, pink denotes those unable to move without assistance, and light blue denotes those who recovered from scabies. N, nurse station; R, recreation room (for occupational therapy); area enclosed by a dashed line, Western style room with wooden flooring. Slight relocation of individuals is not shown.

Figure 3 shows the spatial distribution of scabies cases in the ward with time. The ward is composed of 10 patient rooms arranged in a T-shape with a nurse station at the centre. No restriction of movement or occupational therapy (i.e., recreation activities) was performed to prevent worsening of disturbed cognitive function. For three months from the beginning of the outbreak, occurrence was localized to the tatami mat room where the index case was located. Thereafter, the disease spread to rooms at each end of the ward.

### Disease characteristics

Five patients (25.0%) received treatment with corticosteroid prior to diagnosis. Only 60% (n = 12) complained of an itching sensation, and 4 patients (20.0%) showed difficulty sleeping at night. Major infected areas included the abdomen (n = 9; 45.0%), chest (n = 8; 40.0%), back (n = 6; 30.0%), femoral region (n = 5; 25.0%) and axillaries (n = 5; 25.0%). On the other hand, the neck (n = 1; 5.0%) and inguinal region (n = 1; 5.0%) were rarely affected.

**Figure 4:**
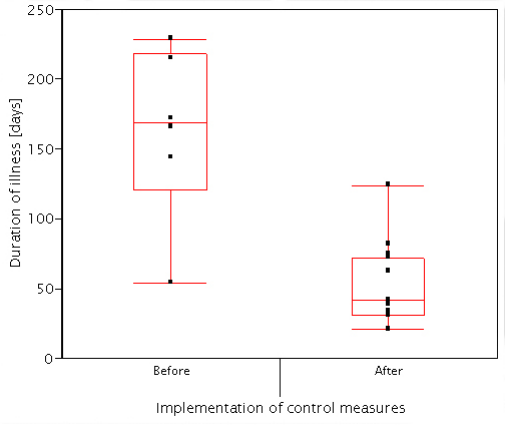
Comparative distributions of the duration of illness after implementation of control measures.

Three patients (15.0%) received treatment with N-Ethyl-N-o-tolylcrotonamide ointment (Crotamiton; Eurax^®^) [6,7], and combination therapy with 1% γ-BHC (1,2,3,4,5,6-hexachlorocychlohexane; Lindane) [8] and Eurax was performed for the remainder (Note: presently, scabies is best treated with oral ivermectin) [9,10]. The mean duration of illness was 85.0 days (SD = 64.3, median = 63.0), and this gradually shortened among later cases (Figure 2). Figure 4 shows distributions of the duration of illness before and after implementation of control measures. Since there was a significant difference in variance between the groups investigated (F = 4.51, df (degree of freedom) = 5, df = 13, P = 0.0264), Welch analysis of variance testing was performed. The mean duration of the first 6 cases (163.3 days) was significantly longer than that of the later 14 infections (53.6 days) (F Ratio = 16.52, t Ratio = 4.06, df = 5.97, P = 0.0067).

### Dementia-specific characteristics

Table 1 summarizes the scabies cases grouped according to investigated exposure factors, and compares non-infected (n = 45) and infected inpatients (n = 20) in each grouping. There was no significant sex-related difference in infection, and types of room and sleeping arrangement were also unassociated with this scabies outbreak. Those able to move without assistance had a significantly higher chance of infection than those who could not (P = 0.0001). Further, movement beyond the room was associated with risk of disease (P = 0.0136).

**Table 1:** Univariate analysis: senile dementia-specific risk factors related to scabies, and comparisons between infected and non-infected individuals

	Scabies cases (n = 20)	Non-infected individuals (n = 45)	p-value^$^	Odds ratio^† ^(95% CI)*
**Gender (male)**	2	6	NS^§^	0.7 (0.1 – 4.0)
**Type of room (Japanese style with tatami mats)**	19	42	NS	1.4 (0.1 – 13.9)
**Sleeping arrangement (on futons directly on the floor)**	18	37	NS	1.9 (0.4 – 10.1)
**Ease of movement (without assistance)**	17	15	0.0001	11.3 (2.9 – 44.8)
**Range of movement (outwith the room but within the ward)**	12	12	0.0136	4.1 (1.4 – 12.5)

^$^Two-tailed; ^†^Odds ratio of being infected through exposure; *95% Confidence interval; ^§^NS: not significant.

## Discussion

We conducted an epidemiologic investigation of a previous scabies outbreak based on individual records and personal observations. The outbreak involved 20 patients with senile dementia. Ward-based control measures were not facilitated until 4 months after diagnosis of the index case, contributing to the prolonged duration of the outbreak of almost 10 months. Although the duration of illness might be biased due to the delay in diagnosis, especially at the early stage of the outbreak, the duration significantly decreased in the latter phase. Using the findings, we attempted to identify dementia-specific risk factors of scabies transmission.

One important conclusion drawn from our study is that a high proportion of the scabies cases were able to move freely around the ward, with those able to move without assistance and outwith their rooms at significant risk of infection. Some cases with cognitive dysfunction were seen walking around the ward yet no specific restrictions were enforced by the HCWs. For example, the patients frequently visited room 106 for no particular reason other than perhaps because it was located at the end of the hallway. It should be noted that transmission of scabies within dementia wards is frequently observed not only among individuals housed in the same or neighbouring rooms but also among those located far from the index case. Since spatial spread enhanced by the behaviour of dementia patients is therefore suggested, our findings support a previous suggestion that transmission through objects (fomites) is far less common [11,12]. A practical dilemma in this ward, however, is that strict control of movement, such as restricted attendance of recreation therapy, is likely to worsen dementia-associated symptoms, and cases in which isolation has triggered mental disturbance have been observed [13]. Patients with dementia tend to have non-specific contacts that are sometimes unpredictable, and consequently, real-time observations of behavioural patterns and individual control measures during daily activities should be among the goals of control practice.

For a long time, senile dementia patients have been known to be at high risk of scabies [1,4,14] and Japan is not an exception [15]. Moreover, scabies among the elderly is known to accompany underlying diseases, causing clinical signs to sometimes last longer than in younger individuals [16]. Another practical dilemma of dealing with this population is that patients with cognitive disorders sometimes do not understand even the chemotherapy treatment they are undergoing [13]. In addition, as observed here, it is extremely difficult to identify probable contacts retrospectively among patients who have difficulty communicating. In addition to discussions on biological risks previously documented in Japan [17], this epidemiologic study suggests that behaviour- and cognitive function-specific characters also play a role in the spread of this disease.

In this outbreak, scabies-specific management among staff members was delayed in the early phase of the outbreak. Furthermore, awareness of infection control practices was insufficient, preventing even chemoprophylaxis. Consequently, no specific restrictions of movement of infectious individuals were made by the HCWs, and thus the dementia patients themselves, whose contacts were unpredictable, contributed to spatial spread. Nevertheless, as a result of careful precautions at a later stage, the observed outbreak eventually declined to extinction. Another difficulty in controlling outbreaks is that it is extremely rare for geriatric hospitals to have an attending dermatologist [16]. As with this outbreak, it seems common to keep the outbreak information confidential. Thus, sufficient institutional and administrative support should be provided to enhance hospital-based control practices.

## Conclusion

This study documented a ward-scale scabies outbreak that lasted for almost 10 months. Initiation of control measures was delayed and, as a result, spatial distribution of cases showed no localized patterns in the later phase of the outbreak. Movement of individuals was identified as a dementia-specific risk factor, while type of room and sleeping arrangement were not significantly associated with infection. Dementia patients tend to have non-specific contacts that are sometimes unpredictable, indicating the necessity of real-time observations of patients' behaviours. Given the requirements of identifying specific risk factors of scabies in dementia wards, we believe our study partly emphasizes the need to enhance and establish hospital-based control practices in long-term care facilities [18].

## Competing interests

The author(s) declare that they have no competing interests.

## Authors' contributions

MT carried out direct investigations of the outbreak, proposed and outlined the study, and was in charge of data handling. HN contributed to the study design and performed statistical analyses. MT and HN reviewed and drafted the manuscript. TK reviewed and commented on the early version of the manuscript. All authors have read and approved the final manuscript.

## Pre-publication history

The pre-publication history for this paper can be accessed here:



## Supplementary Material

Additional file 1 **Case descriptions **Details of individual clinical courses and discussions of management proposals are provided in pdf format. Click here for file
