# Supplementary material for: Dementia-specific risks of scabies: Retrospective epidemiologic analysis of an unveiled nosocomial outbreak in Japan from 1989–90
Source: BMC Infect Dis. 2005 Oct 14;5:85. doi: 10.1186/1471-2334-5-85 (PMC1276794; doi:10.1186/1471-2334-5-85)
Supplement: Additional file 1 — Case descriptions Details of individual clinical courses and discussions of management proposals are provided in pdf format. [file 1471-2334-5-85-S1.doc]

# Additional file1

# Dementia-specific risks of scabies: Retrospective epidemiologic analysis of an unveiled nosocomial outbreak in Japan from 1989-90

# Clinical descriptions of individual courses and features of scabies cases with senile dementia

## Case 1 (85 y/o, female, room 102, duration: 6 May to 19 Oct 1989)

## Movement: within the room, without assistance.

This 85-year-old woman developed red papular eruptions on her abdomen and both femoral regions on 1 May 1989. She was diagnosed with scabies on 6 May afterwhich treatment was performed using 1% γ-BHC (1, 2 , 3 ,4, 5, 6 - hexachlorocyclohexane) ointment. Prior to onset, treatment with corticosteroid for dry eczema had been administered since 4 March. She was observed crawling about on the tatami mats (traditional Japanese mats made of straw and measuring roughly 180 × 90 cm) and walking around the ward, leaning against the walls. She complained of slight itching and slept relatively well during the night.

It was suspected that the application of corticosteroid, which lasted for 2 months, contributed to the prolonged duration of this scabies infestation. Considering the use of steroids and the fact this was the index case of nosocomial transmission, this patient is thought to have had Norwegian scabies. However, there was no evidence of numerous mites and it was difficult to confirm this conclusion by the presently available clinical information alone.

## Case 2 (79 y/o, female, room 102, duration: 3 June 1989 to18 Jan 1990)

## Movement: within the ward (beyond the room), without assistance.

This 79-year-old woman, who shared a room with the index case (case 1), exhibited papular eruptions on her abdomen and both femoral regions in early June, 1989. Diagnosis of scabies was made on 3 June afterwhich she received treatment with 1% γ-BHC ointment. In September, she complained of severe itching and often took off her clothes to scratch herself; however, she was able to sleep deeply. Bean-sized persistent nodes were observed on her back when her treatment schedule was terminated. Despite gradual loss of sight due to glaucoma, she frequently moved about inside and sometimes outside of her room. She always asked someone to be beside her and sometimes slept on futons belonging to other inpatients.

## Case 3 (73 y/o, female, room 102, duration: 17 June 1989 to 18 Jan 1990)

## Movement: within the ward (beyond the room), without assistance.

This 73-year-old woman was admitted on 8 July 1988 and exhibited a dark pachydermatous region in mid-June 1989. Treatment with corticosteroid was instituted to relieve her severe itching, and on 17 June 1989, treatment with 1% γ-BHC ointment was initiated under suspicion of scabies. Numerous ruddy edematous papules were observed on her chest but nothing similar was found on her lower body. From the beginning of September to the end of October her night sleep was disturbed by itching paresthesia. She shared a room with cases 1 and 2 and could walk within the ward without assistance.

Due to the inspecificity of the eruptions, this case of scabies was the most difficult to diagnose.

## Case 4 (82 y/o, female, room 102, duration: 15 July 1989 to 3 Jan 1990)

## Movement: within the ward (beyond the room), without assistance.

Rice-grain sized erythematous patches gradually developed on the armpits and both inner femoral regions of this 82-year-old female in early July 1989. On 15th July, treatment with Eurax ointment was initiated under suspicion of scabies. She complained of mild itching paresthesia. She shared a room with cases 1-3 and frequently crawled about on the tatami mats within the ward. Therefore, exposure to the mites and subsequent infection most likely occurred through physical contact enhanced by her active movement.

## Case 5 (88 y/o, female, room 102, duration: 26 Aug to 19 Oct 1989)

## Movement: within the ward (beyond the room), without assistance.

This 88-year-old woman complained of itching paresthesia on her back at the beginning of August 1989, and frequently requested caregivers and roommates scratch her back. Rice-grain sized erythematous patches developed on her back and armpits, but disappeared after treatment with 1% γ-BHC ointment. After completing treatment, the scratching stopped and she enjoyed deep sleep as a result. She shared a room with cases 1-4 and was observed walking within the ward without assistance.

People with senile dementia generally have difficulty in describing their condition; nevertheless, in this case, oral communication helped the patient receive early treatment and shorten the duration of illness.

## Case 6 (73 y/o, female, room 110 (and 100), duration: 26 Aug 1989 to 17 Jan 1990)

## Movement: within bed, with assistance.

A small rash was observed on this 77-year-old female on 22 July 1989. This coincided exactly with her transference to the ward from another hospital. No information about her skin problem was given by the caregivers of the previous institution. She exhibited papular eruptions on her chest, hip and both femoral regions, and diagnosis of scabies was made on 26 August. During the symptomatic period, she was observed scratching her femoral regions, and on one occasion she scratched her abdomen during the night. When treatment was terminated, it was confirmed that the rash on her abdomen had developed into persistent bean-sized nodes. The patient was initially admitted to tatami-floored room 110 but was transferred into western-style room 100 following fracture of her left clavicle.

This case highlights that careful communication and transfer of clinical information should be performed when a person with suspected scabies is transferred from one institute to another.

## Case 7 (91 y/o, female, room 106, duration: 21 Sep 1989 to 23 Jan 1990)

## Movement: within the ward (beyond the room), without assistance.

This 73-year-old woman was observed frequently crawling down the hallway, sometimes reaching the room at the opposite end (Room 100). She attended group occupational therapy (recreation) almost every day. She exhibited dark pachydermatous skin, similar to case 3, and complained of severe itching. From the end of October to the beginning of November, she would take off her clothes to scratch herself. A small red rash was observed on her chest, both armpits and inguinal regions, and numerous scratch marks were visible.

It was very difficult to make a diagnosis of this patient for reasons similar to case 3. It is believed that this actively moving patient probably distributed *Sarcoptes scabiei* at least within her room. On the other hand, based on our observations, it is also possible that other cases with scabies visited the room resulting in transmission, since this room is located at the dead-end of the hallway.

## Case 8 (84 y/o, female, room 106, duration: 21 Sep to 23 Nov 1989)

## Movement: within the room, without assistance.

At the beginning of September 1989, this 84-year-old female developed ruddy papular eruptions on her back, both armpits and arms. Treatment was initiated using Eurax ointment under suspicion of scabies. Prior to onset, treatment with corticosteroid for dry eczema had been administered since July 1989. The rash did not spread to other regions and treatment with corticosteroid was restarted on 2 November. Since the sensation of itching was quite severe, she frequently scratched herself, and bleeding from her scratch marks was sometimes observed. She was one of the roommates of case 7.

## Case 9 (94 y/o, female, room 106, duration: 28 Sep to 1 Nov 1989, and 18 Jan to 18 Feb 1990)

## Movement: within the ward (beyond the room), without assistance.

This 94-year-old female exhibited ruddy bean-sized papular eruptions on her abdomen and hips at the end of September 1989. On 28 September, empirical treatment with Eurax ointment was started, but eight days later, mites and eggs were still detected on the dry skin of her abdomen and therefore revised treatment with 1% γ-BHC ointment was initiated. On 1 November, she was classified as cured, but on 18 January 1990 mites and eggs were again confirmed in a rash on her chest. Treatment with 1% γ-BHC ointment was therefore restarted. She shared a room with cases 7 and 8, and was observed crawling down the hallway at night.

To prevent and eliminate a scabies outbreak, comprehensive real-time treatment of all exposed individuals is known to be the best strategy. However, in this case, this measure was not put into practice and as a result recurrent infection occurred.

## Case 10 (80 y/o, female, room103 (and 106), duration: 5 Oct to 16 Nov 1989)

## Movement: within the room, without assistance.

This 80-year-old female developed scattered small erythematous patches on her chest and abdomen, which she frequently scratched along with interdigital areas of both hands. She was observed sliding around the room on her back, snuggling up to her roommates and touching their futons. She was therefore transferred to another room (106) when it was suspected that she had scabies, and as a result, no other patients in room 103 developed scabies.

## Case 11 (79 y/o, female, room 102, duration: 19 Oct to 30 Nov 1989)

## Movement: within the ward (beyond the room), without assistance.

This 79-year-old female was a roommate of early cases 1-5. She was observed walking around the ward during the daytime and was unable to keep still due to her cognitive disorder. While walking, she frequently carried a pillow on her back. When asked, “Why are you carrying a pillow?”, she always answered, “This isn’t a pillow, its my baby!” It was also noted that she sometimes touched futons other than her own, and she mimicked taking care of the other inpatients. Rice-grain sized papular eruptions appeared on her back and mites and eggs were detected on 19 October 1989, the date on which she undertook dermatological examinations. Treatment with 1% γ-BHC ointment was performed and the rash disappeared by the beginning of November.

It is sometimes noted that females with dementia tend to handle pillows and cotton blankets for no particular reason. It is possible that the daily habits of their previous lifestyles might be reflected in this behavior. Transmission via fomites is far less common and was not indicated in our epidemiologic study, while movements such as seen in this case can be a risk factor.

## Case 12 (87 y/o, male, room 107, duration: 19 Oct to 21 Dec 1989)

## Movement: within the room, without assistance.

This 87-year-old man developed rice-grain sized papular eruptions mostly on his neck and chest. Treatment with Eurax ointment was instituted on 19 October 1989 under suspicion of scabies. Since mites and eggs were detected on his hip on 26 October, he received treatment with 1% γ-BHC ointment. By the beginning of November, the rash had almost completely disappeared. Thereafter, corticosteroid ointment was applied to the persistent nodes on the abdomen. This patient was observed crawling about within his room but never entering the hallway. Room 107 admitted males only. This case was the first male patient to show typical manifestations of scabies.

## Case 13 (78 y/o, male, room 107, duration: 26 Oct 1989 to 16 Jan1990)

## Movement: within the ward (beyond the room), without assistance.

This 78-year-old man was one of the roommates of case 12. He frequently crawled about the ward carrying his pillow, and sometimes lay down with his head on the pillow in front of room 100, located at the end of the hallway. He had a rice-grain sized rash, mostly on his chest and both arms. He received treatment with Eurax ointment from 26 October 1989 and with 1% γ-BHC ointment from 2 November. A few persistent nodes were observed on his chest but they disappeared one and half months later after treatment with corticosteroid.

The patient probably scattered mites within room 107 before onset of his illness. This is supported by the fact that case 12, who was also infected, never left the room without assistance.

## Case 14 (76 y/o, female, room 110, duration: 26 Oct 1989 to 7 Jan 1990)

## Movement: within the ward (beyond the room), without assistance.

This 76-year-old female was observed walking about within the ward, and was the first case diagnosed in room 110. She exhibited rice-grain sized papular eruptions around her right shoulder and breast at the end of October 1989. Treatment with Eurax ointment was initiated on 26 October under suspicion of scabies. At the beginning of the symptomatic period, she often complained of severe itching, and would exclaim, “Itchy!!” However, she refused treatment in the middle of November for two reasons: relief from the itchy sensation, and loss of communication due to deafness and dementia. Her rash did not spread to other regions; however, a dry area 3 cm in diameter gradually appeared on her neck. This dryness disappeared two months later after treatment with corticosteroid.

A leading cause of treatment failure among patients with senile dementia is that they sometimes understand little about the treatment modalities being offered.

## Case 15 (81 y/o, female, room 110, duration: 2 Nov 1989 to 16 Jan 1990)

## Movement: within the ward (beyond the room), without assistance.

This 81-year-old woman, who shared a room with case 14, exhibited miliary erythematous patches, redness and claw marks on her right shoulder and right upper arm. Diagnosis of scabies was made on 2 November 1989 and a combination therapy of 1% γ-BHC with Eurax ointment was initiated. During the first 2 weeks, she seemed to feel a severe sensation of itching; however, she refused treatment when her rash disappeared, except for a few erythematous areas on her right upper arm. It is unlikely that her futon enhanced the chain of transmission since she was very nervous and used to disallow anyone from touching her belongings. Marks were localized on her right side because of hemiplegia; therefore, she used to scratch using her left hand only. Since she had a speech defect, she communicated her sensation of itching by wiggling her body. This highlights that caregivers should always be watchful for non-verbal appeals of this sort.

## Case 16 (69 y/o, female, room 106, duration: 9 Nov to 18 Dec 1989)

## Movement: within the ward (beyond the room), without assistance.

This 69-year-old woman was a roommate of cases 7 and 10. She was able to walk with a firm step and seemed to have purpose while moving. At the beginning of November 1989, four bean-sized papular eruptions appeared on her abdomen, and gradually another rash appeared on her back. The rash on her back formed into a cluster of miliary-sized eruptions. On 9 November, treatment with 1% γ-BHC ointment was initiated. The rash on her back disappeared quickly but the rash on her abdomen persisted for a while, eventually turning into a few dark red persistent nodes. Corticosteroid was applied to these nodes.

## Case 17 (98 y/o, female, room 108, duration: 9 Nov to 21 Dec 1989)

## Movement: within the room, without assistance.

On 2 November 1989, this 98 year-old woman was transferred to the ward from another hospital. After admission, small red papular eruptions were observed on her entire body except for her head, and on admission, a family member informed the caregivers about her prior skin trouble. They were informed that some kind of mite might have caused the rash, and believed that a certain ointment was applied empirically. However, no information about the rash was received from the previous hospital, as with case 6. Empirical treatment with Eurax was started immediately under suspicion of scabies. Seven days after initiating treatment, diagnosis of scabies was made and treatment with 1% γ-BHC ointment was initiated. Eventually, the rash disappeared completely.

## Case 18 (71 y/o, female, room 111, duration: 30 Nov to 21 Dec 1989)

## Movement: within the ward (beyond the room), without assistance.

Involuntary movement of all four limbs due to degeneration of the basal ganglia was constantly observed in this 75-year-old woman. She was the first patient to be diagnosed in room 111. She was sometimes observed crawling along the hallway, moving without any particular purpose. Four rice-grain sized papular eruptions were observed, three on her abdomen and one on her right femoral region. On 30 November, treatment with 1% γ-BHC was initiated and the rash disappeared soon afterwards. She complained of slight itching only and slept well during the night.

## Case 19 (89 y/o, female, room 100, duration: 7 to 28 Dec 1989)

## Movement: within bed, with assistance.

This 89-year-old woman was located next to case 6 and always stayed in bed. She exhibited numerous small papular eruptions from her left armpit to chest at the beginning of December 1989, and three other eruptions were also observed on her abdomen. On 7 December, treatment with 1% γ-BHC ointment was initiated, soon afterwhich the rash disappeared.

## Case 20 (74 y/o, female, room 105, duration: 7 Dec 1989 to 18 Feb 1990: transferred to another ward where she died immediately)

## Movement: within bed, with assistance.

This 74-year-old woman always stayed in bed, as seen with case 19. On 7 December 1989, bean-sized ruddy papular eruptions suddenly appeared on her chest and abdomen, and a large number of mites and eggs were detected in these regions. Treatment with 1% γ-BHC ointment was instituted immediately, and two weeks later, combination therapy of 1% γ-BHC with Eurax ointment was performed because the rash persisted. Once again, many mites and eggs were detected in inter-digital regions. Her sensation of itching seemed severe since she was always seen scratching during her waking hours. The patient could not be cured, probably for the following reasons: 1) she had been treated with corticosteroid for dry eczema since 29 September 1989; 2) she was diagnosed with diabetes mellitus. However, her fasting blood sugar was controlled in a range of 60-85 mg/dl during the time she was ill, and her HbA1c datum was 3.9 % (31 January 1990); 3) her nutritional status was slightly debilitated, her total protein being 5.0 g/dl on 7 December 1989 and 5.4 g/dl on 10 January 1990; 4) her skin status was generally unclean: she took a bath only four times during more than 70 days of illness; and 5) she was unable to complain about her discomfort due to the decline in her mental faculties.

# Supplementary Discussion

Clinical descriptions of 20 cases were provided highlighting age, sex, room number, range of movement, necessity of assistance for movement, duration of illness, activities of daily life and the course of the disease. The documented signs and symptoms were more or less unusual even among elderly individuals with dementia (i.e., a rather high proportion of nodular reactions and papular lesions on the chest and back were observed). Although it is difficult to confirm by means of epidemiologic methods, based on clinical information it is suspected that the patients might have been infested for longer than measured and were perhaps diagnosed at a rather late stage of illness.

Patients with senile dementia tended to move out of their rooms, while some patients who were basically unable to walk moved actively in other ways such as by crawling. They tended to favor physical contacts with those who provided them with mental support or made them feel calm. Some female patients frequently carried their pillows, probably reflecting their previous habits of daily life. Most patients had difficulty in describing their exact problems verbally.

Caregivers should understand such specific individual characteristics and plan management and treatment accordingly. To prevent scabies transmission, management based on appropriate original observations as well as evidence-based practice is required. Inter-hospital communications should also be improved. Further, a comprehensive approach for treatment of all persons exposed to scabies is desirable; this did not occur in this outbreak. People with senile dementia should be spared the suffering of scabies with appropriate cures and care based on adequate information.
